# *De Novo* Pyrimidine Biosynthesis Connects Cell Integrity to Amphotericin B Susceptibility in *Cryptococcus neoformans*

**DOI:** 10.1128/mSphere.00191-16

**Published:** 2016-11-16

**Authors:** Dithi Banerjee, Timothy C. Umland, John C. Panepinto

**Affiliations:** a Department of Microbiology and Immunology, Witebsky Center for Microbial Pathogenesis and Immunology, University at Buffalo, The State University of New York, Buffalo, New York, USA; b Department of Structural Biology, Hauptman Woodward Medical Research Institute, University at Buffalo, The State University of New York, Buffalo, New York, USA; Duke University Medical Center

**Keywords:** amphotericin B, cell wall integrity, pyrimidine biosynthesis

## Abstract

The use of amphotericin B (AmB) in conjunction with 5-fluorocytosine (5-FC) is known to be the optimal therapy for treating cryptococcosis, but the mechanism by which 5-FC synergizes with AmB is unknown. In this study, we generated a *Cryptococcus neoformans ura1*Δ mutant lacking dihydroorotate dehydrogenase (DHODH), which demonstrated temperature-sensitive growth due to a defect in cell integrity and sensitivity to cell wall-damaging agents. In addition, sensitivity to AmB was greatly increased. Inclusion of uracil or uridine in the medium did not suppress the cell wall or AmB phenotype, whereas complementation with the wild-type *URA1* gene complemented the mutant phenotype. As a measure of membrane accessibility, we assayed the rate of association of the lipid-binding dye 3,3′-dihexyloxacarbocyanine iodide (DiOC_6_) and saw more rapid association in the *ura1*Δ mutant. We likewise saw an increased rate of DiOC_6_ association in other AmB-sensitive mutants, including a *ura*^*−*^ spontaneous *URA5* mutant made by 5-fluoroorotic acid (5-FOA) selection and a *bck1*Δ mutant defective in cell integrity signaling. Similar results were also obtained by using a specific plasma membrane-binding CellMask live stain, with cell integrity mutants that exhibited increased and faster association of the dye with the membrane. Chitin synthase mutants (*chs5*Δ and *chs6*Δ) that lack any reported cell wall defects, in turn, demonstrate neither any increased susceptibility to AmB nor a greater accessibility to either of the dyes. Finally, perturbation of the cell wall of the wild type by treatment with the β-1,6-glucan synthase inhibitor caspofungin was synergistic with AmB *in vitro*.

**IMPORTANCE** Synergy between AmB and nucleotide biosynthetic pathways has been documented, but the mechanism of this interaction has not been delineated. Results from this study suggest a correlation between uridine nucleotide biosynthesis and cell integrity likely mediated through the pool of nucleotide-sugar conjugates, which are precursor molecules for both capsule and cell wall of *C. neoformans*. Thus, we propose a mechanism by which structural defects in the cell wall resulting from perturbation of pyrimidine biosynthesis allow faster and increased penetration of AmB molecules into the cell membrane. Overall, our work demonstrates that impairment of pyrimidine biosynthesis in *C. neoformans* could be a potential target for antifungal therapy, either alone or in combination with AmB.

## Abstract (precis)

Synergy between AmB and nucleotide biosynthetic pathways has been documented, but the mechanism of this interaction has not been delineated. Results from this study suggest a correlation between uridine nucleotide biosynthesis and cell integrity likely mediated through the pool of nucleotide-sugar conjugates, which are precursor molecules for both capsule and cell wall of *C. neoformans*. Thus, we propose a mechanism by which structural defects in the cell wall resulting from perturbation of pyrimidine biosynthesis allow faster and increased penetration of AmB molecules into the cell membrane. Overall, our work demonstrates that impairment of pyrimidine biosynthesis in *C. neoformans* could be a potential target for antifungal therapy, either alone or in combination with AmB.

## INTRODUCTION

The pathogenic fungus *Cryptococcus neoformans* is associated with fatal meningitis, most frequently infecting patients with AIDS or other immune defects resulting from transplantation or chemotherapy (1). The recommended antifungal regimen for the treatment of cryptococcosis is 2 weeks of amphotericin B (AmB) in combination with 5-fluorocytosine (5-FC), a pyrimidine analogue, followed by fluconazole monotherapy (2–4). Studies of fungal burden in cerebrospinal fluid (CSF) in patients with cryptococcosis demonstrate a clear enhancement of the fungicidal activity of AmB in combination with 5-FC compared to AmB alone or AmB in combination with fluconazole (5, 6). *In vitro* work demonstrates synergy between AmB and 5-FC as well as between AmB and mycophenolic acid, an inhibitor of the IMP dehydrogenase in the *de novo* purine biosynthesis pathway (7, 8). The exact mechanism by which AmB and 5-FC synergize is still unknown. The nucleotide biosynthesis pathway is therefore an interesting avenue for further research, with emphasis on its role in potentiation of AmB efficacy.

Dihydroorotate dehydrogenase (DHODH) is a component of the pyrimidine biosynthesis pathway. Two families of DHODH enzymes exist in evolution, family 1 and family 2 (9). Family 1 enzymes are cytosolic and utilize a cysteine as the catalytic residue in the active site (10, 11). Family 2 enzymes, in contrast, are localized to the mitochondrial membrane and contain a serine as the catalytic residue in the active site (10). DHODH enzymes are known to be targetable by small molecules but exhibit enough variation through evolution that species specificity can be achieved in molecule design (12). Brequinar is a known inhibitor of the human DHODH and a family 2 enzyme and is used in immunosuppressive therapy for transplant rejection (13, 14). The malaria parasite also expresses a family 2 enzyme, but the malarial DHODH is not sensitive to brequinar (12, 15), indicating the species specificity of this enzyme. Impairment of *de novo* pyrimidine biosynthesis by perturbing other enzymes in the pathway impairs cell integrity in *C. neoformans* and prevents capsule biosynthesis in the absence of exogenous uracil (16, 17).

In this study, we used a *Cryptococcus neoformans* var. *grubii* DHODH mutant, a *ura1*Δ strain, as a tool to investigate the mechanism by which perturbations in pyrimidine biosynthesis lead to potentiation of AmB fungicidal activity. Deletion of *URA1* resulted in cell integrity defects, temperature sensitivity, mitochondrial defects, replication stress sensitivity, and hypersensitivity to AmB. Rates of association of the membrane-binding dyes 3,3′-dihexyloxacarbocyanine iodide (DiOC_6_) and CellMask green were increased in the *ura1*Δ mutant, suggesting that loss of *de novo* pyrimidine biosynthesis increases membrane accessibility. DiOC_6_ and CellMask green association rates were also increased in a *C. neoformans bck1*Δ mutant, lacking the mitogen-activated protein (MAP) kinase kinase kinase (MAPKKK) in the cell integrity MAP kinase pathway, which was also found to be AmB sensitive. Two chitin synthase mutants, however, did not display increased membrane accessibility to the lipophilic dyes and exhibited wild-type AmB sensitivity. Our results have led us to propose a mechanism by which perturbation of *de novo* pyrimidine biosynthesis impairs cell integrity, thereby allowing increased access of AmB to the cell membrane, resulting in increased susceptibility.

## RESULTS

### *C. neoformans* encodes a single family 2 DHODH required for wild-type growth.

**FIG 1:**
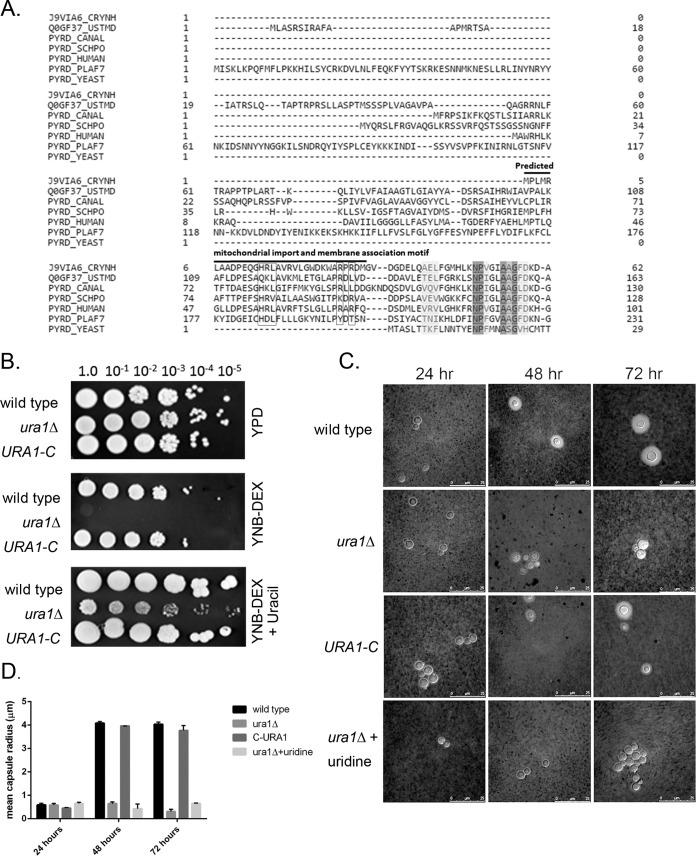
*C. neoformans* encodes a single family 2 DHODH required for wild-type growth. (A) Multiple sequence alignment of *C. neoformans* Ura1 protein sequence (top line) with other eukaryotic family 2 DHODHs including (from top to bottom) *Ustilago maydis* (DQ869012)*, Candida albicans* (AY230865),* Schizosaccharomyces pombe* (NM_001018748), human (NM001361), and *Plasmodium falciparum* (AB070244), and the family 1 enzyme from *S. cerevisiae* (P28272) (bottom line), with only the respective N-terminal regions shown. The first 25 amino acids are important for mitochondrial import and membrane anchorage. The boxes highlight the hydrophobic residues that make up the hydrophobic patches in the family 2 DHODHs. (B) Growth of wild-type, *ura1*Δ mutant, and complemented strains in rich medium (YPD) or minimal medium with and without uracil supplementation. (C) India ink staining of *C. neoformans* capsule under inducing conditions in the wild type, *ura1*Δ mutant, complemented mutant, and *ura1*Δ strain supplemented with uridine at 24, 48, and 72 h. (D) Mean capsule sizes of wild type, *ura1*Δ mutant, *ura1*Δ mutant supplemented with uridine, and complement strain.

*Cryptococcus neoformans* dihydroorotate dehydrogenase belongs to the family 2 DHODHs (Fig. 1), which include the enzymes largely from mammals, higher eukaryotes, and prokaryotes as well (10, 12, 18–20). Family 2 DHODH enzymes from mammals and higher eukaryotes typically possess N-terminal extensions that facilitate their mitochondrial localization and association with mitochondrial membrane. On the other hand, this N-terminal extension tends to be absent in prokaryotic family 2 DHODH, and the protein is bound to the cytoplasmic membrane, in contrast to family 1 enzymes, which are cytosolic (9, 10). Further traits distinguishing DHODH families are their electron acceptor cofactors. Family 1 DHODH uses either a fumarate or NAD^+^ cofactor, whereas family 2 DHODH employs ubiquinone binding in a hydrophobic channel near the N terminus (21). Finally, serine replaces cysteine of the family 1 DHODH enzymes as the catalytic residue and is conserved among all the family 2 DHODH enzymes (10).

Studies from humans, *Plasmodium falciparum,* and other fungal species, including *Candida albicans*, *Schizosaccharomyces pombe,* and *Ustilago maydis,* demonstrate the structure of DHODH enzymes to be comprised of two domains, an N-terminal alpha-helical domain and a large C-terminal domain connected by an extended loop (9, 15, 18, 22). The predicted annotation of the *C. neoformans* var. *grubii* DHODH (CNAG_02794) appears to lack the N-terminal extension present in all other eukaryotic family 2 DHODH enzymes analyzed (Fig. 1A), with an N terminus similar in length to that of prokaryotic family 2 DHODH (data not shown). Our own 5′ rapid amplification of cDNA ends (RACE) data confirm the annotated transcriptional start site. Superimposition of the *C. neoformans* sequence on the structure of human DHODH revealed an absence of a portion of this N-terminal helical region in *C. neoformans*. Despite this truncation, the cryptococcal N-terminal sequence bears a predicted mitochondrial targeting signal peptide (iPSORT prediction tool) and contains the protruding hydrophobic patch with identical positively charged residues (His14, Arg15, Leu16, Arg19, Arg28, and Arg30) (10). The remaining residues important for cofactor binding, substrate binding, and catalytic activity are completely conserved in the *C. neoformans* DHODH sequence (Fig. 1A), including the catalytic serine that is a hallmark of family 2 DHODH enzymes in contrast to the catalytic cysteine in family 1 enzymes (10).

We generated a deletion mutant in the *C. neoformans* var. *grubii* strain H99, a fully virulent and melanizing derivative of H99 (23). We then introduced the wild-type *URA1* gene in *trans* to generate a complemented mutant. Deletion of *URA1* resulted in uracil auxotrophy that was only partially remediated by the addition of uracil and/or uridine to the medium (Fig. 1B). Reintroduction of the wild-type *URA1* gene completely restored uracil prototrophy and wild-type growth. This result suggests that pyrimidine salvage is insufficient for wild-type growth of *C. neoformans*.

The production of the polysaccharide capsule draws from nucleotide pools, as each carbohydrate monomer is added from a nucleotide-sugar conjugate (24, 25). *De novo* pyrimidine biosynthesis is required for capsule production in the absence of exogenous uracil. Consistent with these reports, the *ura1*Δ mutant was defective for capsule production both at 37°C (data not shown) and at 30°C (Fig. 1C). The addition of uracil or uridine did not result in capsule synthesis in the acapsular *ura1*Δ strain (Fig. 1C). Capsule induction was observed after 24, 48, and 72 h of incubation, and the mean capsule size of each strain was tabulated as a bar graph (Fig. 1D), which indicated that capsule induction took place between 24 and 48 h of incubation and that there was no change in capsule size from 48 to 72 h. The fungicidal effect of AmB is fast-acting, as killing is seen within the first 6 to 12 h of exposure by time-kill assays (26–28). Thus, the capsule is likely not contributing significantly to the susceptibility of *C. neoformans* to AmB.

### DHODH is required for wild-type cell integrity.

**FIG 2:**
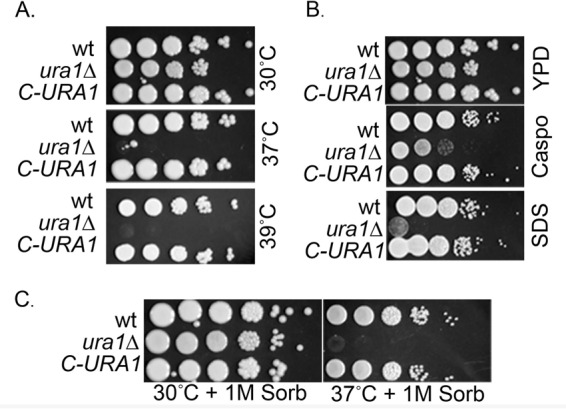
DHODH is required for wild-type cell integrity. Spot plate analysis of WT, *ura1*Δ mutant, and complemented strains on rich medium at the indicated temperatures (A), in the presence of cell wall stressors (B), and in the presence of added osmostabilant (C). Caspo, caspofungin; Sorb, sorbitol.

The cell wall of *C. neoformans* is made up of five polysaccharides, including β-1,6-glucan, α-1,3-glucan, β-1,3-glucan, chitin (α-1,4-GlcNAc), and chitosan (deacetylated α-1,4-GlcNAc). Because each monomer in these large polysaccharide polymers is derived from a UDP-sugar conjugate, it follows that defects in pyrimidine biosynthesis should result in impaired cell wall synthesis (24). To assess the cell integrity of the *C. neoformans ura1*Δ mutant, we performed spot plate assays in the presence of known cell integrity stressors, including the detergent SDS and the β-1,3-glucan synthase inhibitor caspofungin. As demonstrated in Fig. 2B, the *ura1*Δ mutant exhibited sensitivity to the stressors tested, and the reintroduction of the *URA1* gene restored wild-type sensitivity.

Cell integrity is inextricably linked to temperature sensitivity, and so we compared the ability of the *ura1*Δ mutant to grow on rich medium at 30°C, 37°C, and 39°C with those of the wild type and complemented mutant (Fig. 2A). As expected, the *ura1*Δ mutant was sensitive to growth at 37°C and was unable to grow at 39°C. This result is consistent with known attenuation in virulence of *C. neoformans* pyrimidine biosynthesis mutants. To investigate the link between the temperature sensitivity and cell integrity defects of the *ura1*Δ mutant, we assayed the ability of the osmostabilant sorbitol to rescue temperature-sensitive growth in the *ura1*Δ mutant. Figure 2C demonstrates that sorbitol was unable to rescue the temperature-sensitive growth phenotype of the *ura1*Δ mutant, suggesting that additional factors beyond cell integrity contribute to its temperature sensitivity.

### Mitochondrial function and replication stress resistance are impaired in a *ura1*Δ mutant.

**FIG 3:**
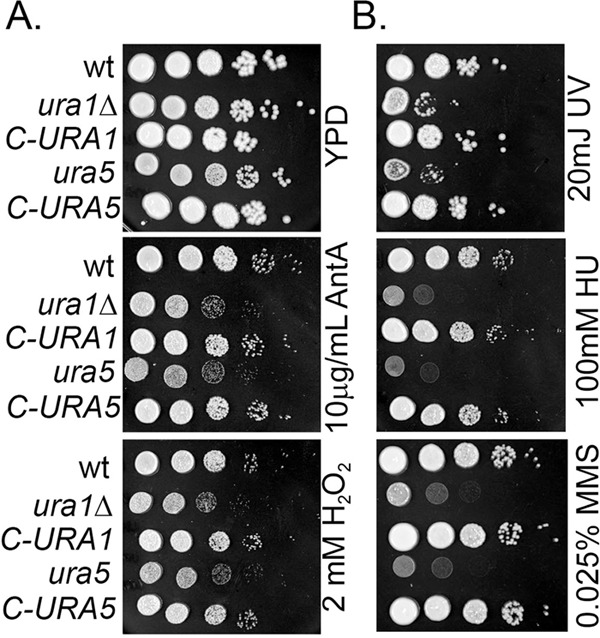
Mitochondrial function and replication stress resistance are impaired in a *ura1*Δ mutant. Spot plate analysis of WT, *ura1*Δ mutant and *C-URA1*, and *ura5* mutant and *C-URA5* strains with mitochondrial stressors (A) and replication stress inducers (B). AntA, antimycin A.

It is known that mitochondrial function is induced in *C. neoformans* in response to host temperature (29). DHODH is a mitochondrial enzyme, and depletion of DHODH in mammalian cells induces mitochondrial dysfunction (30, 31). To determine if the *ura1*Δ mutant exhibited mitochondrial defects, we assayed sensitivity to compounds that perturb mitochondrial function, including the respiratory chain inhibitor antimycin A and oxidative stress inducer H_2_O_2_. Figure 3A demonstrates that the *ura1*Δ mutant was sensitive to mitochondrial perturbation. To determine if this phenotype was unique to the *ura1*Δ mutant or was shared with other *de novo* pyrimidine biosynthesis mutants, we assayed the sensitivity of a spontaneous *ura5* mutant and its complement in parallel. Figure 3A demonstrates a shared sensitivity to mitochondrial perturbation, suggesting that the defects stem from lack of flux through the pathway rather than just the physical absence of the DHODH enzyme.

The homeostasis of nucleotide pools is important for genome integrity in eukaryotic cells. Because pyrimidine salvage is insufficient to fully rescue *ura1*Δ mutant phenotypes, we went on to determine if the *ura1*Δ mutant was sensitive to replication stress. Figure 3B demonstrates that both a *ura1*Δ and a *ura5* spontaneous mutant are deficient in their response to replication stress, including alkylation by treatment with methyl methanesulfonate (MMS), inhibition of ribonucleotide reductase by hydroxyurea (HU), or induction of DNA cross-links by exposure to UV light. Each phenotype was restored completely by reintroduction of the wild-type gene.

### DHODH is required for virulence in a *Galleria mellonella* infection model.

**FIG 4:**
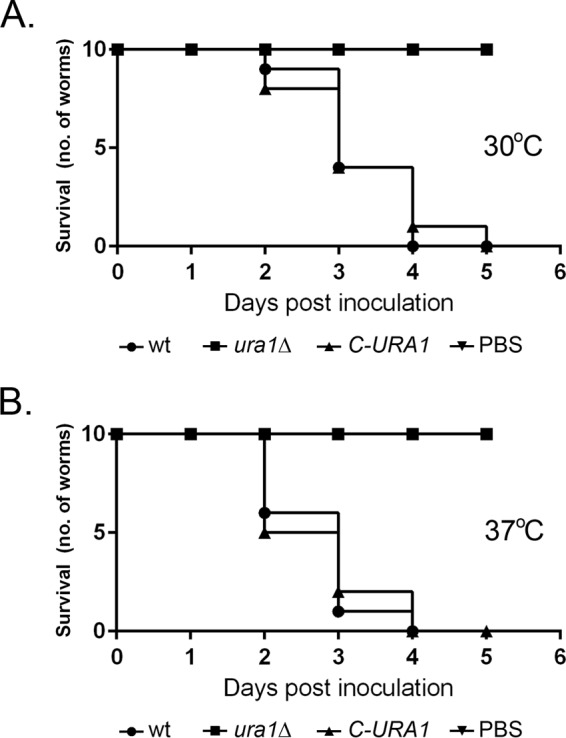
DHODH is required for virulence in a *G. mellonella* infection model. *G. mellonella* larvae were inoculated with WT, *ura1*Δ, and *C-URA1* strains and monitored for survival at both 30°C (A) and 37°C (B).

The larvae of *Galleria mellonella* are a powerful surrogate model for assessing cryptococcal pathogenicity, especially as it relates to temperature adaptation (32). To investigate the contribution of *ura1*Δ mutant temperature sensitivity to pathogenesis, we compared the abilities of the wild type, the *ura1*Δ mutant, and its complement to cause mortality in larvae incubated at either 30°C or 37°C. Figure 4 demonstrates a rapid killing of *G. mellonella* larvae by the wild-type H99 strain and *URA1*-complemented strains in comparison to the *ura1*Δ mutant, indicating the requirement of DHODH for virulence in an infection model of cryptococcosis. Wild-type and complemented strains killed all the larvae by day 4 at both 37°C (Fig. 4B; P < 0.05) and 30°C (Fig. 4A; P < 0.05), whereas 100% survival was observed in larvae infected with the knockout strains at both temperatures. These data demonstrated that in addition to temperature sensitivity, pyrimidine pool depletion also plays a role in virulence, similarly to results obtained with a *ura4*Δ mutant (16). No death was observed in any of the mock-injected controls at either 30°C or 37°C until 5 days postinoculation.

### Loss of DHODH sensitizes *C. neoformans* to amphotericin B.

**FIG 5:**
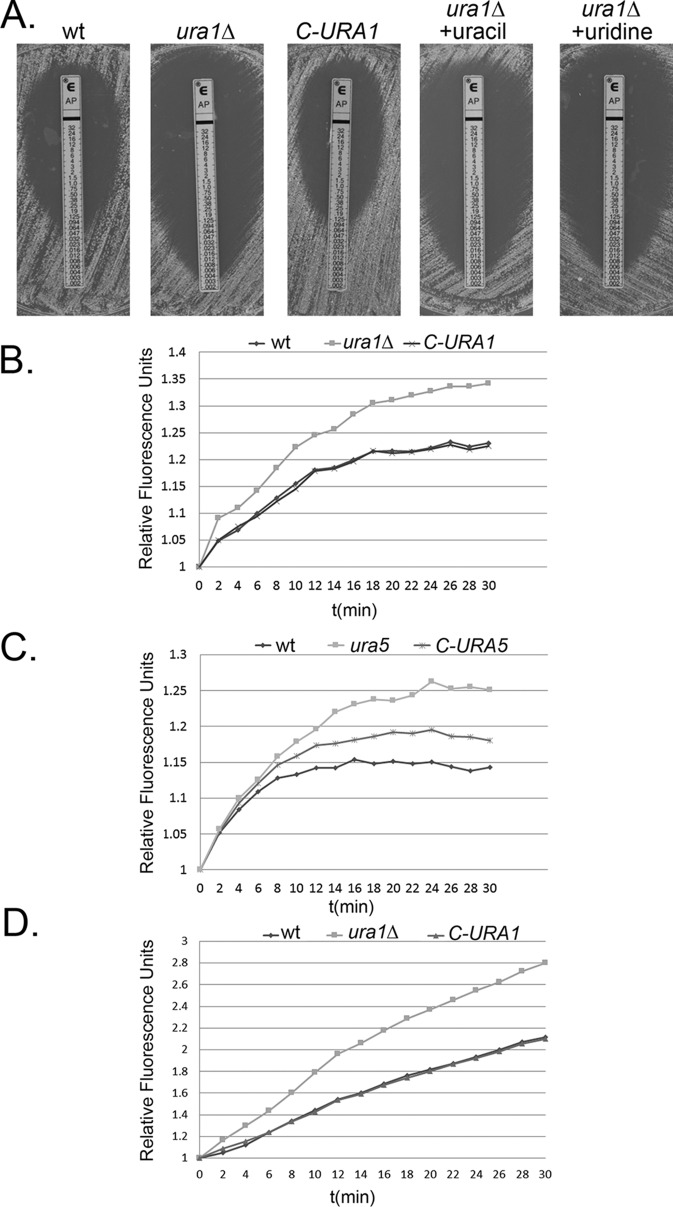
Loss of DHODH sensitizes *C. neoformans* to amphotericin B. (A) Etest analysis of AmB sensitivity in the wild type, *ura1*Δ mutant, and complemented strain on AM3 medium alone or with the *ura1*Δ mutant in the presence of uracil or uridine. (B and C) Comparison of the rates of association of DiOC_6_ stain between the *ura1*Δ mutant (B) or the *ura5* mutant (C) and the wild type or the respective complemented strains. (D) Comparison of the rates of association of CellMask green live stain between the *ura1*Δ mutant, wild type, and complemented mutant.

Perturbations in *de novo* pyrimidine biosynthesis by mutation or treatment with nucleoside analogues potentiate the fungicidal activity of AmB. To investigate the AmB sensitivity of the *ura1*Δ mutant, we performed both Etest and broth microdilution MIC assays. As expected, the *ura1*Δ mutant was found to be sensitive to AmB with a MIC of 0.004 µg/ml (Fig. 5A), similar to that of a spontaneous *ura5* mutant selected on 5-fluoroorotic acid (5-FOA) in both assays (17), in comparison to 0.064 µg/ml and 0.0094 µg/ml, the MICs of the wild type and the *C-URA1* mutant, respectively. Etest in the presence of exogenous uracil and uridine also showed lowered MIC levels (0.004 µg/ml) in the *ura1*Δ mutant (Fig. 5A), which suggests that pyrimidine salvage is insufficient to compensate for the higher sensitivity to AmB in the absence of *de novo* synthesis.

The mechanism by which perturbation of pyrimidine biosynthesis potentiates AmB activity is unknown. A simple hypothesis is that the cell integrity defects induced by inhibition or mutation of *de novo* synthesis allow increased access of small molecules to the cell membrane. To test this hypothesis, we compared the rates of association of the membrane-binding dye DiOC_6_ with the wild-type, *ura1*Δ mutant, and complemented strains (33). As demonstrated in Fig. 5B, the fluorescence intensity of the *ura1*Δ mutant increased more rapidly and to a higher equilibrium than that of either the wild type or complemented mutant, suggesting that the cell wall of the *ura1*Δ mutant allowed for more rapid penetration and membrane binding of DiOC_6_ than of either the wild type or the complemented mutant. We assessed if this phenotype was shared with the spontaneous *ura5* mutant made by 5-FOA selection and found an increase in the rate of DiOC_6_ association similar to that of the *ura1*Δ mutant (Fig. 5C). To determine if this increased dye association was indeed a result of faster and easier permeability to lipophilic small molecules, we performed a similar experiment with CellMask green stain, another membrane-binding dye that is specific to the plasma membrane (34). Results demonstrate higher relative fluorescence units (RFU) in the *ura1*Δ mutant over a shorter period of incubation, which suggests a faster accessibility of the dye to the plasma membrane in the mutant compared to either the wild type or complement (Fig. 5D).

### Cell integrity defects correlate with AmB sensitivity.

**FIG 6:**
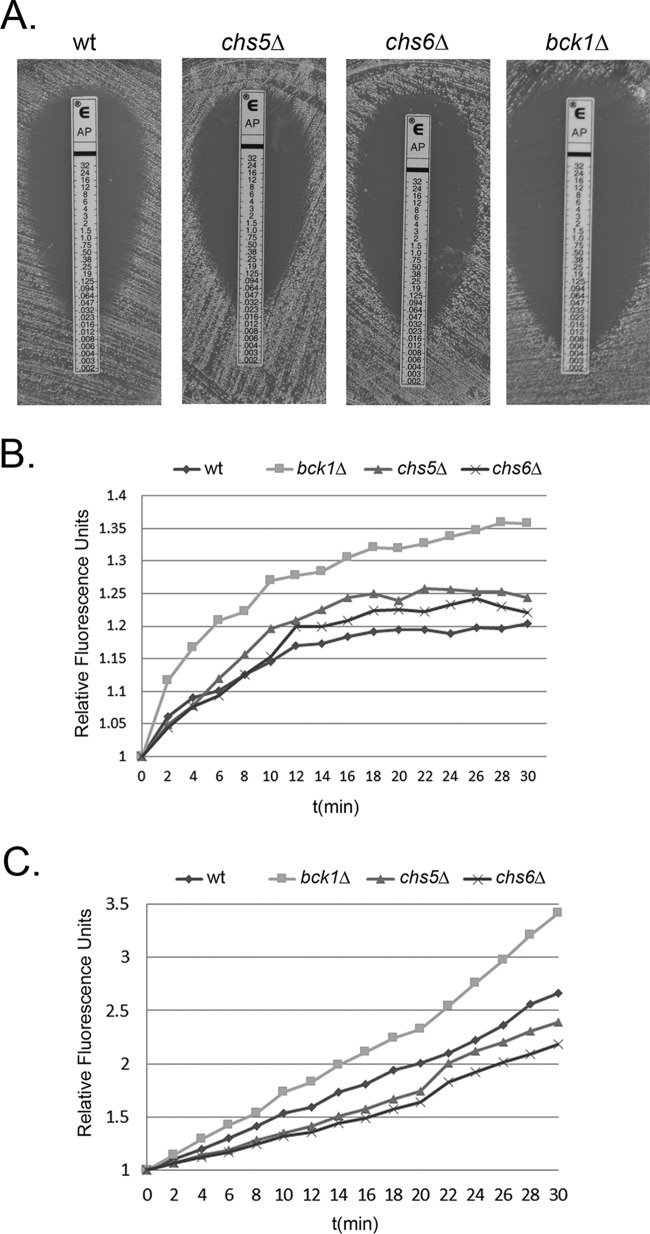
Cell integrity defects correlate with AmB sensitivity. (A) Etest analysis of AmB sensitivity in the wild type, *chs5*Δ mutant, *chs6*Δ mutant, and *bck1*Δ mutant. (B and C) Comparison of the rates of association of DiOC_6_ stain (B) and CellMask green live stain (C) between the wild type, *chs5*Δ mutant, *chs6*Δ mutant, and *bck1*Δ mutant strains.

If the mechanism of AmB potentiation in the *ura1*Δ mutant is increased access to the membrane, we would expect that other mutants with cell integrity defects would exhibit sensitivity to AmB. We selected a panel of cell integrity mutants from the Lodge Lab collection obtained through the Fungal Genetic Stock Center (http://www.fgsc.net). The selected mutants were *bck1Δ* (lacking the MAPKKK in the cell integrity MAP kinase cascade) and *chs5*Δ and *chs6Δ* (mutants of chitin synthase genes *CHS5* and *CHS6*, respectively) strains. We performed Etests and broth microdilution assays to determine the MIC of AmB for each of the strains. Results revealed an increased sensitivity of the *bck1Δ* mutant (MIC, 0.004 µg/ml), whereas the *chs5Δ* and *chs6Δ* mutants demonstrated wild-type MIC levels of 0.047 µg/ml (Fig. 6A). The result from the *bck1Δ* strain suggested that this increased sensitivity to AmB could be due to the defect in cell integrity, consistent with our hypothesis (35). We know that there are eight *CHS* genes and they are redundant in function, except for *CHS3* (36). Previous studies have demonstrated notable phenotypes in only the *chs3Δ* strain and mild phenotypes in *chs6Δ* knockout strains. We believe that *chs5Δ* and *chs6Δ* mutants do not have significant cell integrity impairment and thus exhibit normal sensitivity to AmB. To verify this hypothesis, we also performed DiOC_6_ and CellMask green staining with these strains to investigate their membrane accessibility to the dyes. Results in Fig. 6B and C demonstrated faster and greater permeativity of DiOC_6_ and CellMask dyes, respectively, in the *bck1Δ* mutant and wild-type dye binding capacity in the *chs5Δ* and *chs6Δ* mutants that correlated with our findings from the AmB MIC assays. Taken together, our data suggest that a defect in cell integrity potentiates the antifungal efficacy of AmB.

### Perturbation of cell wall synthesis also potentiates AmB fungicidal activity.

**FIG 7:**
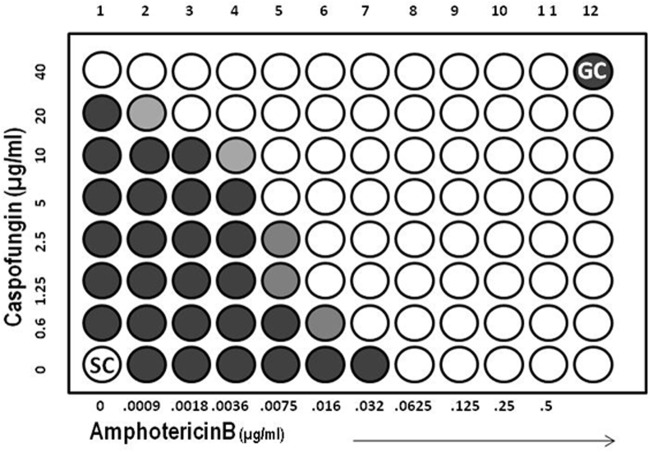
Perturbation of cell wall synthesis also potentiates AmB fungicidal activity. Checkerboard analysis of the activity of AmB and caspofungin in combination against wild-type *C. neoformans*.

Finally, because cell integrity is linked to AmB susceptibility, we expected that inhibition of cell wall synthesis by the β-1,3-glucan synthase inhibitor caspofungin would lead to potentiation of AmB activity. To test this, we quantified the interaction between caspofungin and AmB by checkerboard MIC analysis (37, 38). Results demonstrated in Fig. 7 indicate that the mean MIC of AmB alone from three independent experiments was 0.06 µg/ml and that of caspofungin alone was 40 µg/ml. Mean MIC was significantly lowered by approximately 3-fold to 0.0075 µg/ml in AmB and by 4-fold in caspofungin to 10 µg/ml. The fractional inhibitory concentration (FIC) index calculated from the MIC values to determine the drug interaction is reported in Table 1. An FIC value of <0.5 indicates synergy, a value ranging between 0.5 and 1.0 indicates additive interaction, any value between 1.0 and 4.0 is suggestive of indifference, and a value of >4 indicates antagonism (39). We found that a combination of caspofungin and AmB was synergistic in action with an FIC index of 0.375, and this result further suggests that a defect in cell wall synthesis potentiates the antifungal activity of AmB.

**TABLE 1:** FIC index of AmB and caspofungin drug interaction in wild-type *C. neoformans*

Drug or parameter	Concn (µg/ml)		
	Replicate 1	Replicate 2	Replicate 3
AmB alone (MIC_A_)	0.06	0.06	0.06
AmB in combination (MIC_A′_)	0.0075	0.0075	0.0075
Caspofungin alone (MIC_C_)	40	40	40
Caspofungin in combination (MIC_C′_)	10	10	10
FIC index = MIC_A′_/MIC_A _+ MIC_C′_/MIC_C_	0.375	0.375	0.375
Interaction	Synergy (≤0.5)	Synergy (≤0.5)	Synergy (≤0.5)

## DISCUSSION

Polyene antifungals exert their activity through interactions with ergosterol in the fungal cell membrane (40). The functional consequence of this interaction is thought to be pore formation and cell disruption, although other modes of action have been proposed. The potentiation of AmB activity by 5-FC is known, and inclusion of 5-FC in combination with AmB is the recommended initial therapy for treatment of cryptococcosis. The use of AmB in underresourced areas of the world is hampered by its toxicity (41). Defining the underlying mechanism of this synergy could inform future drug design to improve the activity of AmB and possibly reduce the required dose to achieve effective antifungal activity. Reduced dosage could improve safety and widen availability for use of this potent antifungal drug.

DHODH is a known druggable target. Brequinar and leflunomide target the mammalian DHODH (14, 21). Compounds that specifically target the malarial DHODH are currently in clinical trials (42). The *C. neoformans* var. *grubii* DHODH is distinct from other eukaryotic family 2 DHODH enzymes in that the hydrophobic channel produced by the N-terminal alpha-helices is truncated. This makes *C. neoformans* DHODH more interesting since this trait is shared by prokaryotic family 2 DHODHs. Because this region corresponds to the binding region of known inhibitors, it is unlikely that the *C. neoformans* enzyme will be effectively inhibited by molecules that target other fungal family 2 enzymes. *C. neoformans* is insensitive to brequinar, an inhibitor of the human DHODH (data not shown). This is unsurprising, as the closely related basidiomycete *Ustilago maydis*, which encodes a family 2 DHODH with the conserved N-terminal extension, is also resistant to brequinar (18). The resistance in these fungi is likely not due to permeability of the drug, as replacement of the *U. maydis* DHODH with the human enzyme results in brequinar sensitivity.

The utility of inhibiting *de novo* pyrimidine biosynthesis in organisms with intact salvage pathways has been questioned. However, studies from *C. neoformans* demonstrate that *de novo* synthesis of pyrimidines is required for virulence in animal models of infection (16, 43). *C. neoformans* is unique among the pathogenic fungi in that it generates a large polysaccharide capsule that, in addition to the cell wall polysaccharide, utilizes nucleotide sugar monomers in its production. Thus, the salvage pathway of nucleotide biosynthesis alone may not be sufficient to support the construction of these polymers in addition to maintenance of genome integrity and RNA synthesis in the absence of *de novo* synthesis. Our time course data as well as the investigation of capsule regulators suggest that there is no correlation between capsule production and AmB susceptibility. Both acapsular and hypercapsular regulatory mutants display varied patterns of susceptibility to AmB (35, 44–46).

Inhibition of *de novo* pyrimidine biosynthesis does impair cell wall synthesis and mitochondrial function, both of which are required for adaptation to host temperature. Increased temperature induces mitochondrial function and results in oxidative damage of important proteins. We have shown in Fig. 3A that loss of DHODH sensitizes *C. neoformans* to the mitochondrial stressor antimycin A and the oxidative stressor hydrogen peroxide, suggesting that this protein is required to protect the cells from oxidative damage. This leads us to believe that in the absence of DHODH, oxidative stress, in addition to cell integrity defects, contributes to the temperature sensitivity of the *ura1*Δ mutant. Thus, an inhibitor of *de novo* pyrimidine biosynthesis would impair two additional distinct pathways required for pathogenesis. Our data using the *Galleria* model of infection demonstrate that temperature sensitivity alone cannot account for the decrease in virulence, as equivalent defects in virulence were seen in the larvae when incubated at 30°C and at 37°C. It follows that inhibitors of *de novo* nucleotide biosynthesis should not be discounted in the design of novel anticryptococcal agents.

The data presented in this study support a model in which inhibition of pyrimidine biosynthesis causes defects in the cell wall of *C. neoformans* that, in turn, increase access of small molecules such as AmB to the cell membrane. Thus, small-molecule inhibitors of nucleotide biosynthesis, cell wall synthesis, and cell integrity signaling pathways should uniformly result in increased sensitivity to AmB. In addition, pharmacological agents that are modestly effective in monotherapy, such as caspofungin, could be highly effective in the context of combination therapy.

## MATERIALS AND METHODS

### Strains.

*C. neoformans* strain H99 (serotype A), a *ura5* spontaneous mutant, and the *C-URA5* complement strain were described previously (17). The *bck1*Δ (35) and *chs5*Δ and *chs6*Δ (36) strains were obtained from the deletion collection of Jennifer Lodge through the Fungal Genetic Stock Center at Kansas State University.

*ura1*Δ knockout and *C-URA1* complement strains were produced as follows. A *C. neoformans ura1*Δ strain was constructed as described previously (47). The construct was verified by sequencing, which showed retention of 610 bp of the *URA1* gene as the 5′ flanking sequence. This partial knockout construct was biolistically transformed in wild-type H99 (48), and colonies were screened for uracil auxotrophy by simultaneous serial passage in yeast extract-peptone-dextrose (YPD) broth and minimal yeast nitrogen base (YNB) medium supplemented with 2% dextrose (Dex). The clones that grew in YPD but failed to grow in the absence of exogenous uracil in YNB-2% Dex were verified by Northern and Southern blot analysis. Because pyrimidine-rich tracts that flank the *URA1* gene precluded use of PCR, a complementation construct was isolated by screening a size-selected genomic library. Wild-type H99 genomic DNA was first digested with EcoRI and BamHI (EcoRI and BamHI restriction sites present at 5′ and 3′ flanking regions of the *URA1* gene) to generate a product of 3,547 bp which encompasses the *URA1* gene. This 3.5-kb piece was ligated into pBluescript plasmid, and colonies were selected by blue-white screening followed by colony hybridization probing with the *URA1* coding region. The construct was then transformed into the *ura1*Δ knockout strain by electroporation (49). Transformants were selected on minimal medium lacking uracil, confirming uracil prototrophy. Northern blot analysis confirmed expression of wild-type DHODH. Primer sets used in this study are available from the authors upon request.

### Media and reagents.

*C. neoformans* strains were maintained in yeast extract-peptone-dextrose (YPD; BD Difco) broth supplemented with 80% glycerol (Fisher Chemicals) as frozen stocks and freshly streaked on YPD agar (BD Difco) before experiments. Spot plate assays were performed on YPD agar alone or supplemented with different reagents. Uracil auxotrophy was detected on yeast extract nitrogen base with ammonium sulfate (BD Difco) agar alone or supplemented with 2% dextrose (YNB-2% Dex) or uracil (Sigma-Aldrich), uridine (Sigma-Aldrich), or both. RPMI 1640 (Gibco) was used for capsule induction and staining assays. Antibiotic medium 3 (AM3) (Difco, MD, USA) buffered to pH 7.0 with 10 mM phosphate was used for the Etest, and AM3 supplemented with 2% Dex was used for microbroth MIC detection assays and checkerboard assays.

### Protein structure modeling.

The three-dimensional structure of *C. neoformans* DHODH was predicted via homology modeling using the Swiss-Model server (50). A crystal structure of human DHODH (PDB 2PRL; chain A, residues 30 to 396) was used as the structural template, possessing 43.7% sequence identity to the *C. neoformans* homologue.

### Spot plate assays.

*C. neoformans* wild-type, mutant, and complemented strains were grown as overnight cultures, washed twice, resuspended, and diluted to an optical density at 600 nm (OD_600_) of 1.00 in sterile distilled water. Tenfold dilutions of wild-type H99, *ura1*Δ mutant, *C-URA1* complement strain, *ura5* spontaneous mutant, and *C-URA5* complement cells were plated onto YPD agar plates alone or supplemented with 0.02% sodium dodecyl sulfate (SDS; Invitrogen) and 20 µg/ml caspofungin diacetate (Sigma Aldrich) for investigating cell integrity stress, 5 µg/ml antimycin A (Sigma-Aldrich) and 2 mM hydrogen peroxide (H_2_O_2_; Fisher) for mitochondrial stress, or 100 mM hydroxyurea (HU; Sigma-Aldrich) and 0.025% methyl methanesulfonate (MMS; Sigma-Aldrich) for replication stress and incubated at 30°C for 48 h. Additionally, YPD agar plates were spotted with the control and test strains, irradiated with 20 mJ UV, and incubated at 30°C to investigate replication stress. Inoculated YPD agar plates with and without sorbitol (Fisher) were also incubated at 30°C, 37°C, and 39°C for determining effects with temperature stress.

### Capsule detection assay.

Wild-type, *ura1*Δ mutant, and *C-URA1* complement strains were grown to mid-log phase in YPD. Cells were then harvested, washed twice, resuspended in sterile distilled water, and adjusted to an OD_600_ of 1.0. Two hundred microliters of this cell suspension was inoculated in 5 ml 1× RPMI alone or supplemented with uracil or uridine in a tissue culture plate for capsule induction and incubated at 30°C in 5% CO_2_ for 48 to 72 h. India ink preparations were made as mentioned previously (17) at 24, 48, and 72 h of incubation, and capsule was observed using LAS-AF software (Leica).

### Membrane staining assay using DiOC_6_ and CellMask live stain.

Cells (10^5^) from overnight cultures of wild-type (WT), mutant, and complement strains were washed and resuspended in phosphate-buffered saline (PBS; Gibco), mixed with 200 nM DiOC_6_ (Life Technologies, Inc.) stain or 100× CellMask stain (Thermo Fisher) for membrane staining. Cells were incubated in 96-well black-bottomed Greiner plates, and readings for relative fluorescence units (RFU) over 30 min of incubation were taken by a spectrophotometric plate reader (SpectraMax M5; Molecular Devices, CA) with excitation at 482 nm and emission at 504 nm for DiOC_6_ and excitation at 485 nm and emission at 535 nm for CellMask green live stain.

### MIC detection by Etest.

Cells (0.5 × 10^7^) of wild-type, *ura1*Δ mutant, and *C-URA1* complement strains were plated onto AM3 alone or supplemented with 20 µM uracil or 20 µM uridine, and *bck1*Δ, *chs5*Δ, and *chs6*Δ strains were grown on AM3 alone as a lawn culture with a sterile cotton swab. Plates were dried completely, after which amphotericin B Etest strips (BioMérieux) were placed in the center of the plates. Following 48 to 72 h of incubation at 30°C, the MIC was determined for each of the strains by a standard method (17).

### MIC detection by broth microdilution method.

Suspensions of wild-type, *ura1*Δ mutant, and *C-URA1* complement colonies were made in sterile distilled water, washed three times, and diluted to yield a final concentration of 1 × 10^7^ cells. Working solutions of amphotericin B (Cellgro; Mediatech, Inc.) were made in AM3 at double the concentration that was used in the assay. One hundred microliters of increasing concentrations of the drug was dispensed in the 96-well microtiter plate followed by 100 µl of the inoculum to reach a final volume of 200 µl. Final concentrations of AmB ranged from 0.0009 µg/ml to 0.5 µg/ml in the assay. A well containing 100 µl each of assay medium and inoculum served as growth control (GC), whereas a single well containing 200 µl of the assay medium alone served as sterility control (SC). The plate was incubated at 37°C for 48 to 72 h, after which readings were recorded spectrophotometrically using an automated plate reader set at 540 nm. The MIC endpoint for AmB was determined as the lowest drug concentration where there was complete inhibition of growth.

### Checkerboard analysis.

To determine the drug interaction between AmB and caspofungin, working solutions of each drug were prepared in AM3-2% Dex at a concentration four times the targeted final concentration in the assay range. One hundred microliters of drug mixture (50 µl of increasing concentrations of one drug and 50 µl of fixed concentration of the other drug) was mixed and dispensed in the 96-well microtiter plates. Final concentrations of antifungals for MIC detection ranged from 0.0009 µg/ml to 0.5 µg/ml and 0.6 µg/ml to 40 µg/ml for AmB and caspofungin, respectively. Wells were designated GC and SC as explained above. One hundred microliters of yeast inoculum (with final cell concentration of 1 × 10^5^ CFU/ml) was dispensed in each well except the SC, following which trays were incubated at 37°C for 72 h. Readings were recorded spectrophotometrically. The MIC endpoint for AmB was determined as explained above, whereas for caspofungin, the MIC was defined as the lowest concentration of drug tested alone and in combination at which the turbidity in the well was 50 to 80% less than in the control well (GC). The FIC of each drug and the FIC index were calculated from the respective MIC and FIC values to determine the interaction status of the drugs tested (17).

### *Galleria mellonella* killing assay.

*G. mellonella* larvae were allowed to acclimate in the dark at 30°C and 37°C overnight prior to being injected. Ten larvae of required weight (250 ± 25 mg) were selected for each group in the assay. Inoculum was prepared by washing overnight cultures of wild-type, *ura1*Δ mutant, and *C-URA1* cells three times and resuspending them in PBS. Cells were counted in a hemocytometer slide and diluted with PBS to yield an inoculum size of 1 × 10^8^ cells that was injected by a 10-µl Hamilton syringe. Ten-microliter aliquots of the inoculum were injected into the hemocoel of each larva via the last proleg after disinfecting the injected area with an alcohol swab. One group of larvae was injected with PBS to serve as a control to monitor killing due to physical injury. The larvae were incubated in the dark at 30°C and 37°C and monitored daily to record the number of dead larvae. Death was considered when the caterpillars melanized and/or failed to respond to touch. Killing curves were plotted over time to depict survival of the different strains at the two different temperatures.

### Statistical analyses.

GraphPad Prism 6.0 software was used for statistical analyses of capsule sizes using the grouped analyses (two-way analysis of variance [ANOVA]), and standard deviation was plotted on the graph. Kill curves in the *G. mellonella* infection model experiment were analyzed using the Kruskal-Wallis analysis (ANOVA on ranks), and a *P* value of <0.05 was considered significant. Experiments were performed in triplicates.
