# Correction for Banerjee et al., “*De Novo* Pyrimidine Biosynthesis Connects Cell Integrity to Amphotericin B Susceptibility in *Cryptococcus neoformans*”

**DOI:** 10.1128/msphere.00406-23

**Published:** 2023-09-26

**Authors:** Dithi Banerjee, Timothy C. Umland, John C. Panepinto

Volume 1, issue 6, e00191-16, 2016, https://doi.org/10.1128/mSphere.00191-16. Figures 1 and 2 mistakenly contain duplicated images that require explanation, as detailed below. We acknowledge our error and have taken steps to ensure that the results as presented are reproducible. These errors do not impact the conclusions of the article.

Page 3, Fig. 1C: The image for “ura1Δ, 24 h” is not the correct image but is a mistakenly repeated panel from reference 17. It is expected that *Cryptococcus neoformans* strains would exhibit minimal capsule under these conditions, and therefore, the misincorporation of this panel does not change the conclusion of the figure. Furthermore, repeated experiments have reproduced this result.

Page 5, Fig. 2B: The same control plate image as in Fig. 2A was used. The following should be added to the end of the Fig. 2 legend: “Note that the YPD control plate is the same in panels A and B, as the experiment was performed simultaneously and the control panel was duplicated for the purpose of showing the types of stress separately.”

